# Gratitude in Health Care: A Meta-narrative Review

**DOI:** 10.1177/1049732320951145

**Published:** 2020-09-13

**Authors:** Giskin Day, Glenn Robert, Anne Marie Rafferty

**Affiliations:** 1 Florence Nightingale Faculty of Nursing, Midwifery and Palliative Care, King’s College London, London, United Kingdom; 2 School of Medicine, Imperial College London, London, United Kingdom

**Keywords:** gratitude, health care, meta-narrative review, psychology, qualitative

## Abstract

Research into gratitude as a significant sociological and psychological phenomenon has proliferated in the past two decades. However, there is little consensus on how it should be conceptualized or investigated empirically. We present a meta-narrative review that focuses on gratitude in health care, with an emphasis on research exploring interpersonal experiences in the context of care provision. Six meta-narratives from literatures across the humanities, sciences, and medicine are identified, contextualized, and discussed: gratitude as social capital; gifts; care ethics; benefits of gratitude; gratitude and staff well-being; and gratitude as an indicator of quality of care. Meta-narrative review was a valuable framework for making sense of theoretical antecedents and findings in this developing area of research. We conclude that greater attention needs to be given to what constitutes “evidence” in gratitude research and call for qualitative studies to better understand and shape the role and implications of gratitude in health care.

## Introduction

In recent years, gratitude has emerged as a compelling component of psychological and physical well-being (Yoshimura & Berzins, 2017). Research has burgeoned. The surge in attention has been attributed to renewed scrutiny of virtue ethics in moral philosophy (Gulliford et al., 2013), the rise of positive psychology as an academic discipline (McConnell, 2016), and the potential role for gratitude practices in addressing psychopathologies (e.g., Duprey et al., 2018).

Gratitude research is still in the nascent phase of development as a topic—partly because there is no consensus on whether gratitude is primarily a moral quality or whether its value resides in the acts of expression and reception of gratitude. Gratitude has multiple statuses as, among others, an emotion, a character trait, a psychological characteristic, a material gesture, and a politeness response. Accordingly, views diverge on how it should be constructed in theory or approached as a topic for empirical investigation (Gulliford et al., 2013). In the history of ideas, gratitude has been approached from multiple perspectives, including psychology, philosophy, theology, sociology, anthropology, humanitarian studies, and positive organizational scholarship. Drawing on these intellectual traditions, we present a meta-narrative review of current research on gratitude which focuses on the context of health care interactions. We provide a portrait of gratitude research in health care, highlighting areas that have led to new insights and suggesting areas that would benefit from further development.

### Objectives and Focus for Review

The objective of this review—the first meta-narrative review of gratitude in the context of care-giving relationships—is to identify theoretical frameworks that have shaped scholarship in the expression and reception of gratitude to draw out common threads and show areas of divergent thinking. Our focus on health care is predicated on the premise that gratitude is context dependent: values, policies, and practices all shape the ways in which gratitude is expressed, received, welcomed, or withheld. While gratitude can be expressed to inanimate objects (Boleyn-Fitzgerald, 2016), the “standard view” is that gratitude describes an interpersonal relationship in which it is a response to a benefit provided by a benefactor (Shaw, 2013). This justifies our attention to literature that explores gratitude in the context of interpersonal relationships and capacity building within health care.

## Methods

### The Meta-narrative Approach

Meta-narrative literature review is a method for synthesizing and conceptualizing research approaches to topics that have been studied by different groups of researchers (Wong et al., 2013). It is a semi-systematic approach that retains the interpretive engagement, inductive reasoning, and cross-interrogation of the narrative review for which Thorne (2019) has recently advocated. The meta-narrative method, originally proposed and developed by Greenhalgh et al. (2004, 2005), has proved useful for making sense of topics that transcend disciplinary boundaries. The review followed the RAMESES (Realist And MEta-narrative Evidence Syntheses: Evolving Standards) publication standard. The standard outlines the phases that researchers should undertake in planning and executing a meta-narrative review. Guiding principles are pragmatism, pluralism, historicity, contestation, reflexivity, and peer review (Wong et al., 2013).

### Scoping the Literature

The initial process of exploratory scoping of the literature involved thinking broadly about the topic of gratitude and how it manifested in research paradigms within the disciplines with which it has been associated. From this overview, we familiarized ourselves with the way different authors conceptualized gratitude, and which empirical research and theoretical ideas were considered significant by multiple authors. This is analogous to a “territory mapping” exercise (Wong et al., 2013). To assemble the boundaries of the review, we focused on peer-reviewed scholarly journals, requiring included articles to have a discernible aim and findings and/or recommendations in which gratitude was elaborated in the context of health care. Second, gratitude needed to be addressed as a concept in this article, either through an implicit or explicit definition, or situating it within a theoretical framework.

### Search and Selection Process

Three databases, chosen to reflect a range of scholarly sources, were searched from their inception to November 2019: ProQuest includes 23 databases that cover social sciences, arts and humanities, and nursing; PubMed covers journals and books in the life sciences and biomedicine; and Academic Search Complete was chosen because of its multidisciplinary content.

Search strategies were complicated by “gratitude” frequently being used in the acknowledgment sections of articles (e.g., a full-text search of the database ProQuest for “gratitude” reveals nearly 1.5 million documents). Restricting the search to article titles was an effective way of identifying articles that specifically dealt with gratitude as a point of focus for the article. We added the term “healthcare” OR (“health” AND “care”) in the full-text search. A set of 191 articles was returned from this first run of the e-search strategy (June 2019).

Once duplicates had been merged, 160 articles were identified as potentially suitable for inclusion. We screened the articles using the following criteria:

1. Does the article deal with gratitude as a concept?
2. Does the article deal with gratitude in a health care context?
3. Is the article from a source likely to yield substantive content (e.g., peer-reviewed journal rather than newsletter or magazine)?
4. Is there enough substantive content (gratitude is defined, theorized and/or discussed) to be worth analyzing?

Forty-nine articles met these criteria and were initially included in the analysis. However, once data extraction began, it became evident that there was an anomaly in the use of the term “health care” during the sifting phase. An approach that included health care as a setting rather than a practice led to a predominance of articles in the field of health psychology in which many of the articles employed what might be termed “drive-through gratitude”: the inclusion of an instrument—generally the self-report questionnaire Gratitude Questionnaire-Six-Item Form (GQ-6; McCullough et al., 2002)—among a battery of other surveys without adequate justification or conceptual consideration. We therefore placed more emphasis on the *care* part of “health care” so that the relational aspects in which we had a particular interest were afforded sufficient profile. Clinical settings were not a prerequisite for inclusion, but all the included studies involved a therapeutic context (in practice or in professional development) in which gratitude was implicated in care relationships.

The revisiting of sifting criteria 2 and 4 with a critical eye led to a more robust dataset that fulfilled the “pluralism” criterion for a meta-narrative review as identified by Greenhalgh et al. (2005). A further 24 articles were excluded, leaving 25 articles included from the first systematic search. A rerun of the search strategy in November 2019 led to a further seven recently published articles being included. Promising-looking citations were followed-up which, once screened, led to the addition of 24 further articles. A total of 56 studies were included in the final review (the process is summarized in Supplementary Figure 1, and all included articles are given in Supplementary Table 1).

### Data Extraction

The following characteristics were recorded in a data extraction form: aim of the study; definition of gratitude (along with whether this was explicit or implicit); the theoretical underpinnings of the article; academic discipline; whether it was a commentary/editorial, qualitative, quantitative, or mixed methods article; methods used (if any); study setting and participants; whether gratitude was expressed or received; the nature of any gratitude intervention; if quantitative, which instrument was used; the article’s focus; and findings and/or recommendations (Supplementary Table 1).

### Analysis and Synthesis

We independently noted the characteristics that were descriptive of the research traditions, or fields of study, to which we felt the included articles belonged. These were either specific academic disciplines with associated methods (e.g., positive psychology or sociology), or context-driven scholarship (e.g., health education, policy). For articles that were vague about their paradigms and conceptual modeling of gratitude, an examination of implicit definitions and methodological framing helped to align the research within particular traditions.

The meta-narratives were arrived at through independent inductive coding initially and then an iterative process of discussion and review among the authors to refine the list of potential meta-narratives to ones that we most confidently felt described the body of work under review. We took into account the theoretical underpinnings that the articles referred to, key authors or studies cited as informing the article’s approach, and the ways in which findings were framed, paying careful attention to any imagery and metaphors used. Having assigned each article to at least one meta-narrative, we mapped each article’s focus and disciplinary orientations, acknowledging that the characteristics could not be exhaustive and allowing for some articles to fit more than one meta-narrative.

## Results

### Article Characteristics

**Figure1.**
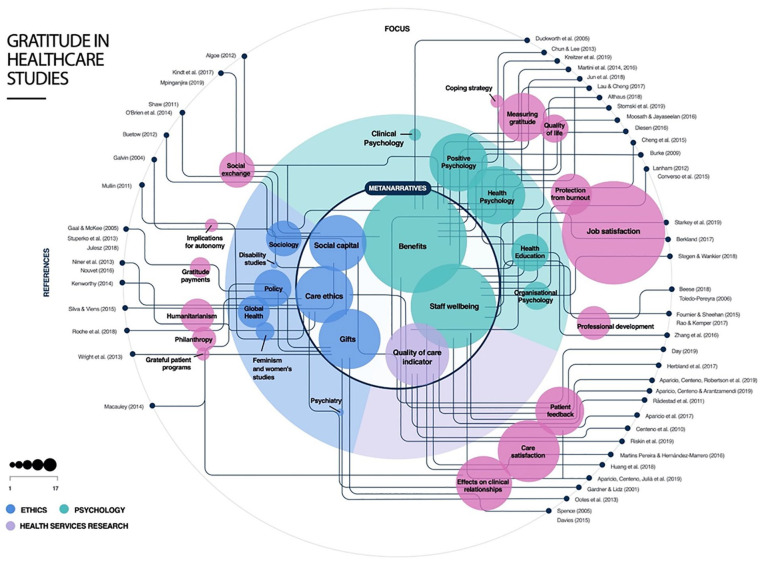
The visual representation of the review

Although no date limits were imposed on the search criteria, all the included articles date from 2000 onwards with most published after 2013. Ten of the included articles were editorials or commentaries, 20 presented qualitative research, 16 quantitative research, and 10 used mixed methods. Of the articles included, 31 gave an explicit definition of gratitude. These most often cited a definition by Emmons and McCullough (2003) or a variation of this in which gratitude is thought of as a generalized tendency to notice and experience appreciation for the good in daily lives or a response to a benefit received. Other characteristics are reported in Supplementary Table 1 and Figure 1.

## Main Findings

The meta-narratives we identified are arranged below according to the chronology of their theoretical antecedents. This gives a sense of the evolution of distinct but related research traditions that have shaped each narrative.

### Meta-narrative 1: Gratitude as Social Capital

The term “social capital” is thought to have first been used by Hanifan (1916) who defined it as assets that “count for most in the daily lives of a people, namely goodwill, fellowship, mutual sympathy and social intercourse among a group of individuals and families who make up a social unit” (p. 130). Since then, sociologists—including those working in health care (see Derose & Varda, 2009 and Jaye et al., 2018)– have made much use of the metaphor of “capital” to refer to intangible qualities, like gratitude, that can be thought of as being accrued or expended in particular circumstances.

Many of the studies included in this meta-narrative reported that the accumulation of social capital through gratitude empowers and motivates recipients through strengthening social bonds, encouraging social connectedness, and predicting willingness to reciprocate. Gratitude as empowering is elaborated in particular in Algoe and Stanton (2012), Buetow and Aroll (2012), Day (2019), Kindt et al. (2017), and O’Brien et al. (2014). However, for those obligated to expend social capital through gratitude for care, autonomy is eroded. The pernicious effects of a grateful consciousness are discussed specifically in Galvin (2004) and Kenworthy (2014)—these two articles are also allocated to the “care ethics” meta-narrative in which the relationship between gratitude and power relations is elaborated more fully.

Two articles, Buetow and Aroll (2012) and Mpinganjira (2019), directly refer to social capital. Buetow and Aroll describe gratitude as a form of social capital which supports “a contribution-based morality” (p. 2064), and that can add “joy and meaning” to a doctor’s work while strengthening social ties. In contrast, Mpinganjira constructs gratitude, not as a form of social capital per se, but as an emotion that mediates the relationship between social capital and willingness to reciprocate. Drawing on the disciplinary perspective of resource-exchange theory, she argues that managers of virtual health communities can instrumentalize gratitude as a strategy to stimulate knowledge sharing on their sites.

This body of research often describes a temporal dimension in which gratitude can be “carried over” from one time point to another. Kindt et al. (2017), for example, use a framework of self-determination theory to contextualize their findings that the partners of chronic pain patients are more motivated to provide help after their partners are perceived as being grateful. Similarly, in a wide-ranging, nuanced analysis of the experiences of heart transplant patients, O’Brien et al. (2014) shows that “giving back by giving forward” is a common phenomenon in which donor recipients often express their gratitude by participating in support groups and research, and through advocacy.

The language of reciprocity, using economic metaphors, features strongly across all the articles in this meta-narrative. The philosopher Claudia Card likens the balance metaphor to “moral bookkeeping” in formulations of gratitude common in moral ethics (C. Card, 1988, p. 116). She explores obligation as a means to understanding gratitude as part of the dynamics of interpersonal relationships, a concept which underpinned many of the articles included in this meta-narrative. Card notes that the debtor paradigm of obligation is a paradox: one cannot repay a debt of gratitude without transforming it into a transaction in which gratitude instinctively has no place. Critiquing moral economics, she maintains that unpayable debts in this paradigm, where reciprocity is not practical or desirable—as is often the case in health care—make the sense of obligation problematically unresolvable. This position is supported by the research we reviewed that engaged with the meta-narrative of social capital: while economics metaphors are prevalent in the discourse of gratitude, the way it plays out in practice in health care is much more psychologically and philosophically subtle than the metaphor of “capital” suggests.

In this meta-narrative, gratitude is construed as a moral incentive to reciprocity, or a persistent “debt” when reciprocity is not possible. Although social capital is intangible, these studies show that it does have material consequences for the dynamics of human relationships and social behaviors. The meta-narrative of gifts, discussed next, is also concerned with reciprocity, but here gifts are tangible: they are the giving and receiving of material goods, physical tokens of appreciation, or—controversially—gifts of money.

### Meta-narrative 2: Gifts

Theory underpinning human behavior in relation to gifts is dominated by Marcel Mauss’s influential 1925 essay on gifts (Mauss, 2000, f.p. as *Essai sur le don*). Mauss argued that gifts are never disinterested: the expectation of return is what consolidates social ties in gift-giving relationships. Gifts are not inevitably associated with gratitude, and gratitude does not demand a gift, but much gift-giving does go on in health care settings and this raises ethical issues (see Drew et al., 1983, for discussion of gifts to doctors, and Morse, 1989, 1991 for gifts to nurses). There is a large literature associated with gift-giving, of which this review includes only those articles on gifts specifically linked to gratitude as a *prima facie* motivation in a health care context. The included articles have in common a focus on the ethical and policy implications of gifts of goods or money presented by patients, either to individual health care providers or to organizations.

Authors that deal with gifts recognize that gift-givers’ motives may be benign if motivated purely by gratitude for care deemed worthy of extra recognition, but gifts become problematic when a gift is given in anticipation of privileged treatment. Spence (2005) and Ootes et al. (2013) draw on psychoanalytic frameworks to explore the mindsets of patients who give gifts. Spence (2005) explores the risks of doctors accepting gifts, urging special caution for gifts that arise “out of the blue” before the doctor has done anything to “deserve” them. Ootes et al. (2013) also urge practitioners to reflect carefully before accepting gifts. In their ethnographic study in a Dutch mental health care context, they identify types of gifts for professionals and discuss these in the context of social inclusion of clients and professional codes. They argue that attention should be paid to gift-giving as potentially altruistic instead of invariably interpreting gifts in terms of reciprocity.

Some gift-giving practices are described as “gratitude” but are, in reality, obligatory cultural norms. There are a number of articles that scrutinize Eastern European customs of giving “gratitude payments” (Gaal & Mckee, 2005; Julesz, 2018; Stepurko et al., 2013). Gratuity payments were usually legal in the 19th century when doctors were paid more than promised for a job well done or received gifts such as produce, wine, or art. During the Communist era, it was the social norm for patients to pay doctors for ostensibly free medical services. Low pay for medical workers has contributed to the persistence of informal payments. In his study of the practice in Hungary, Julesz (2018) found that payments are still customary although they are contrary to the Code of Ethics, and those soliciting money in advance are prosecuted (although low penalties mean that this does not act as a deterrent). The author argues that all such payments are corruption, and says that, alarmingly, “In the post communist part of the world and also in a great many developing African countries, authors always mean corruption when they use the word ‘gratitude’” (p. 157).

The ethics of “cultivated’” gratitude were also explored in Wright et al. (2013) and Macauley (2014). Hospitals often channel donations from grateful patients and their families into philanthropic programs that seem, at first, to circumvent the compromising effects of individuals accepting gifts. But these authors show that these initiatives (sometimes called “grateful patient programs” in the United States) are not immune to exploitative tactics that can compromise trust in the doctor–patient relationship.

The discourse on gift-giving in articles in this meta-narrative often mentions “questions”: unfinalized practices that tend to raise questions to which there are no easy answers. Medical professionals are urged to ask themselves questions about the motivations of patients in giving gifts, necessitating a degree of interpretation that cannot be encoded in policies. Research aligned with this meta-narrative explores tensions between gifts as benign gestures of gratitude that are culturally normative and gifts that are essentially supplementary fees or tips. Issues around the giving and receiving of gifts pose fundamental ethical questions about beneficence and autonomy to which authors of articles in this meta-narrative have been acutely alert.

### Meta-narrative 3: Gratitude and Care Ethics

Articles in which acts of charity or generosity are conceptualized metaphorically as gifts fall outside the boundaries of the way we have circumscribed the gift meta-narrative. These articles engage less with the transaction of goods or money and more with the implications of gratitude as a response to a construal of “care-as-gift.” An example is the study by Kenworthy (2014) which argues global health interventions in developing countries can be interpreted as a gift for which gratitude is the obligatory response. She explores how this engenders “new debts, obligations, and forms of peonage for recipients” (p. 83). Articles like this, which also explore balancing of power and voice, were assigned to our third meta-narrative: gratitude and care ethics.

Care ethics as a field of ethical theory was founded by Carol Gilligan, whose research on relationships between identity and moral development led her to locate care as central to women’s “different voice”—a voice which binds relationship and responsibility, calling for responsiveness and careful listening to voices that were previously met with indifference (Gilligan, 1993). In the context of gratitude, an ethic of care pays meticulous attention to the voices and the circumstances of those expressing gratitude to understand its impulses and implications. These articles were generally characterized by a qualitative, anthropological approach based on in-depth case studies and underpinned by a well-elaborated theoretical framework that drew on Gilligan as well as subsequent work by feminist and disability theorists.

Addressing a feminist ethics of care most directly is Mullin (2011) who argues that gratitude is consistent with how relations of care are understood as morally valuable when they attend to the needs and also the capacities of care recipients. She contests the idea that those who are paid to care are not appropriate targets for gratitude, arguing that gratitude is important in generating mutual respect. In common with Algoe and Stanton (2012), she finds that gratitude is distinguished from indebtedness because motives of goodwill and caring are imputed to the benefactor rather than the expectation of equivalent payback. Acknowledging that both the recipients and the providers of caring labor are groups of people who need support, Mullin says it is important for both care recipients and providers to make sufficient time to demonstrate mutual goodwill and respect, and gratitude is integral to this relationship.

Galvin (2004), however, warns of the problematic nature of gratitude when it exacerbates a lack of autonomy for physically disabled people through ongoing reliance on informal care in which gratitude is the only currency available: “For those who are able-bodied, gratitude may well comprise a comfortable and unproblematic response to kindness, but for disabled people it can signify an unbearable state of perpetual obligation” (p. 137). She found that people who had access to paid personal assistance tended to feel a greater sense of control, comfort, and autonomy than those constrained by feelings of shame and frustration when having to be persistently grateful for the goodwill of others.

A similar wariness is expressed in the study by Niner et al. (2013) of the birthing experiences in Australia of displaced Karen women from Burma. The women they interviewed expressed gratitude for a variety of circumstances (safe haven, secure environment, care given, and post-birth support) in spite of many having experienced suboptimal care and a lack of autonomy, exacerbated by a lack of interpreters. The authors attribute the women’s “gracious acceptance stance” (p. 544) to imperatives to normalize distress in the context of adverse past experiences and their self-reliant attitudes, as well as cultural aversions to complaining.

Consistent gratitude as a hallmark of entrenched disempowerment is similarly a theme considered by Nouvet (2016) who explores the power effects of gratitude in the context of American surgical missions to Nicaragua. Nicaraguans interviewed felt the patient-centered care they received from foreign missions stood in contrast to the dehumanizing, discriminatory treatment they had experienced in the public health care system. While noting the importance of the “small drops of humanity” (tone of voice, vocabulary, smiles) for which many patients expressed gratitude, the author notes the ambiguity of the politics of gratitude in that it simultaneously enacts affirmations and denunciations of the status quo. Similarly, Roche et al. (2018) found that explicit, unprompted gratitude was expressed by nearly all the aid recipients they interviewed in Guatemala. In common with Nouvet (2016), the authors hypothesize that foreign visiting medical teams may unwittingly contribute to inequalities by making ongoing access to benefits contingent on appropriate display of “grateful postures” and that recipients of aid may be construed as failing to successfully navigate and pay within formal health structures.

All of the articles that enact the meta-narrative of a care ethic are attuned to the voices of the grateful, listening to but also interpreting narratives within a framework of politics and power relations. Ambiguity is a key concept here: gratitude is a sincere response to good intentions and care that is often delivered with humanity and warmth. But context is all important. When gratitude becomes obligatory, it moves from being an act of responsive relations to a marker of disenfranchisement and may exacerbate health inequalities.

### Meta-narrative 4: Benefits of Gratitude

Overwhelmingly, the empirical work identified in this review reported on the benefits of being grateful. Although published in a wide range of journals, this work is most often situated in the paradigm of positive psychology—a field of scholarship introduced by Seligman and Csikszentmihalyi (2000). Proponents of positive psychology consciously seek to counter the dominant medical model of human functioning that focused on distress and pathology while neglecting factors that contribute to well-being, happiness, and life satisfaction. In studies allied with this meta-narrative, gratitude has been investigated as a personality disposition, or trait, that is correlated with well-being, possibly with a causal relationship (Wood et al., 2010). Studies sought ways of measuring the beneficial effects of gratitude, elaborating associations with other measures of well-being and life satisfaction, and/or evaluating gratitude interventions like journaling or “counting blessings” exercises.

Attention to the positive effects of gratitude dates from a collaboration between psychologists Robert Emmons and Michael McCullough in the late 1990s. McCullough et al. (2001) offered an influential functional theory of gratitude and reinforced it with empirical support. A landmark edited volume *The Psychology of Gratitude* followed in 2004 (Emmons et al., 2004). The “breakthrough” article that heralded this a new paradigm in empirical gratitude research is McCullough et al. (2002). The authors describe a series of studies that validate a self-report 6-item, unifactorial questionnaire (GQ-6) for measuring trait gratitude, that is, a grateful disposition or character. Eight of the articles in our review used GQ-6 or a modified version of it. Other scales, notably the Gratitude, Resentment and Appreciation Test (GRAT) and the Appreciation Scale, are described and reviewed in Davidson and Wood (2016) and compared in N. A. Card (2019). Martini and Converso (2014) have developed a scale, PGrate, specifically to measure health care providers’ perceptions of patients’ expressions of gratitude. To date, the PGrate scale appears only to have been used by its authors (Converso et al., 2015; Martini & Converso, 2014; Martini et al., 2016).

Articles included in this meta-narrative often focus on gratitude benefits as a factor that could be used instrumentally to inform care interactions. Althaus et al. (2018) conclude that gratitude may have a positive impact on quality of life and reduce psychological distress in patients receiving palliative care in Switzerland. A thematic analysis of interviews with patients in a city in the United States who had suffered a traumatic spinal cord injury found that patients benefited from appraising adverse life experiences as positive through the lens of gratitude (Chun & Lee, 2013).

Studies have investigated gratitude interventions as possible therapies. Kreitzer et al. (2019) found that gratitude practice in an online therapeutic community led to reported improvements in stress levels, gratitude, and social support, although effects were relatively short lived. Moosath and Jayaseelan (2016) analyzed questionnaires and conducted interviews with eight patients receiving chemotherapy in an oncology ward of a Bangalore hospital, India, who took part in a gratitude journaling intervention. The study found that gratitude journaling boosted subjective well-being and also gave insights into patients’ reflections on the nature of gratitude.

Benefits of gratitude were identified, not only for patients, but for familial and professional caregivers. Lau and Cheng (2017) carried out a survey of Chinese familial caregivers of people with dementia and found that gratitude was related to emotion-focused coping and psychological resources that reduced distress. A study by Stomski et al. (2019) of associations between gratitude and carer burden in informal Australian mental health carers had more equivocal results: simple appreciation was associated with a higher care burden, but the trait of “lack of sense of deprivation” (a focus on what a person has) and an appreciation of others reduced the burden leading the authors to recommend that gratitude interventions should specifically target these tendencies.

The metaphor usually associated with the “benefits” meta-narrative is “building.” Positive psychology was described by Duckworth et al. (2005) as a “build what’s strong” rather than a “fix what’s wrong” approach (p. 631), and this imagery is at the heart of one of the most influential models of gratitude, attributed to Barbara Fredrickson: “broaden-and-build.” The model holds that “positive emotions appear to *broaden* people’s momentary thought-action repertoires and *build* their enduring personal resources” (Fredrickson, 2004, p. 147, italics in original). This is in contrast with negative emotions that invoke a narrow thought-action repertoire for quick and decisive action in situations which may be life-threatening. Although the situations which bring forth positive emotions may be transient, Fredrickson argues that the personal resources that one builds are durable and can be drawn on to cope and survive.

Methodological limitations, which have also been discussed in gratitude scholarship more widely (see, for example, Gulliford et al., 2013; Jans-Beken et al., 2018, 2019; Lambert et al., 2009), were evident in the research we reviewed here. Empirical studies tended to report low to modest effect sizes and gave limitations like small sample sizes, narrow sampling bands, high attrition rates in long-term studies, and difficulty in setting up meaningful control groups.

Articles in this meta-narrative approached gratitude as having benefits for psychological well-being of patients and informal caregivers. Patients tended to have long-term or lifelong conditions. Carers, too, who were research participants tended to be involved in familial or long-term caring relationships. It was notable that both populations were seen as being resilient but prone to psychological distress—hence, their potential to benefit from broaden-and-build gratitude interventions. Studies that examined these benefits within a *professional* health care context had different emphases which warranted a separate meta-narrative: gratitude and staff well-being.

### Meta-narrative 5: Gratitude and Staff Well-being

The mental and physical health of health care practitioners is a matter of global concern (see, for example, Cheng et al., 2015, and O’Connor et al., 2018). The meta-narrative of gratitude and staff well-being is concerned with interventions, surveys, and reviews that focus on gratitude expressed or received by health care students and professionals. Although mostly situated within positive psychology, research in occupational therapy, positive organizational scholarship, and health education also informs these studies. They have in common a construction of the professional caregiver as vulnerable to stress and burnout against which gratitude awareness and practice might protect. The cultures of care in professional settings explored by studies in this meta-narrative interrogate the role of gratitude in enhancing job satisfaction, reducing absences, improving retention, and/or boosting teamwork—factors that did not feature strongly in the studies involving informal caregivers that we assigned to the benefits meta-narrative.

Interventions in health care education and professional development encourage participants to express gratitude as a means of enhancing their own well-being but also to augment their capacity for patient- and person-centered care (Fournier & Sheehan, 2015; Rao & Kemper, 2017). Our review includes one randomized controlled trial of a gratitude journaling intervention for health care practitioners across five hospitals in Hong Kong, which found that the practice effectively reduced perceived stress (−2.65 points; 95% confidence interval [CI]: [−4.00, −1.30]; *d* = −0.95) and depressive symptoms−1.50 points; 95% CI: [−2.98, −0.01]; *d* = −0.49; Cheng et al., 2015). The most ambitious of the studies included in this review is by Stegen and Wankier (2018) who implemented multiple gratitude interventions over the course of a year within the nursing faculty at Weber State University, Utah, USA. The authors found that post-intervention survey participants reported that job satisfaction increased, as did teamwork and collaboration. In a wide-ranging study of virtues, work satisfactions and well-being among 79 nurses in a single hospital, Burke et al. (2009) found that nurses scoring higher on gratitude showed more job satisfaction, vigor, dedication, and few absences.

In studies that look at the impact of patients’ gratitude on staff, a scoping review by Aparicio et al. (2019) found that gratitude may have important personal and professional effects on health care professionals. A self-report study of oncology and emergency nurses at two Italian hospitals by Converso et al. (2015) suggests that perceptions of patients’ gratitude could have a protective effect against burnout. Starkey et al. (2019) also found receiving expressions of gratitude predicted physical health benefits in a survey of 146 nurses in Oregon, USA, via satisfaction with patient care.

Imagery that is prevalent in this meta-narrative is that of “levels.” The analyses speak of raising, improving, promoting, or enhancing desirable qualities such as morale and compassion and lowering, reducing, or decreasing or factors perceived as problematic such as stress. One study spoke of examining the impact of various “doses” of skills training (Rao & Kemper, 2017). In common with literature in the benefits meta-narrative, Fredrickson’s broaden-and-build theory was often invoked as an explanatory framework (Fredrickson, 2004).

### Meta-narrative 6: Gratitude as an Indicator of Quality of Care

There is a rich tradition of studying the effects of emotions in social interactions to try to understand helping and cooperative behaviors, and the way the self is evaluated according to the feedback of other social actors (see, for example, Tangney et al., 2007). Although not usually specifically referred to, a hypothesis underlying many of the studies included in this meta-narrative is “feelings-as-information.” This hypothesis, articulated by Norbert Schwarz, holds that affect has cognitive consequences that can influence judgment (Schwarz, 2012). The articles we grouped in this meta-narrative linked gratitude and quality of care. Perspectives were explored either from the patients’ or relatives’ points of view in which gratitude is expressed after an experience of the delivery of good care, or in which gratitude precedes and predicts the delivery of high-quality care.

While the writing of gratitude letters is a common gratitude intervention thought to contribute to the well-being of the writer, the receiving of such letters by health care professionals or institutions is generally regarded as an indicator of quality of care. Several authors have conducted thematic analyses of unsolicited letters to care units to evaluate their usefulness as a form of feedback on care provided and as a source of narratives of the patients’ or relatives’ experience (Aparicio et al., 2017; Centeno et al., 2010; Herbland et al., 2017; Martins Pereira, & Hernández-Marrero, 2016). This meta-narrative is also linked, either explicitly or implicitly, to staff benefits in that there is a perception that access to such letters can boost self-esteem among staff, potentially reducing burnout and acting as a motivating factor for staff. Herbland et al. (2017) also link their study to an ethic of care, arguing that thank-you letters received by the intensive care unit at a French Hospital resonate with phases of care consistent with Gilligan’s characterization of care as a reciprocal practice (Gilligan, 1993).

In a historical study that looked at correspondence between 1,506 former patients with tuberculosis and staff at the Brompton Hospital in London in the 20th century, Day (2019) found that gratitude was central to the ongoing relationships of care that saw many patients continuing to correspond with the hospital for decades after discharge. Day argues that communication strategies that acknowledge and build on gratitude have useful lessons for enhancing relational care in today’s health care settings.

Riskin et al. (2019) also make recommendations for how gratitude can improve care. Their intervention study in Israel found that teams hearing a mother expressing gratitude prior to a simulation exhibited significantly better diagnostic and treatment performance during a neonatal clinical intensive care unit training session. In common with Day (2019), the authors call for better acknowledgment within health care of the positive impact of gratitude gestures.

Two studies, Rådestad et al. (2011) and Diesen (2016), solicited patient or service users’ feedback through a questionnaire and interviews, respectively. Rådestad et al. (2011) analyzed answers to a question about gratitude to staff to argue that changes in care practices in Sweden around 1990, allowing parents increased contact with their stillborn child, were effective. Diesen (2016) found gratitude to be a theme in the reflections of young adults in Norway with phenylketonuria. The authors argue that gratitude could be a major coping strategy for patients, in which a focus on the positive is an active and informed choice.

In articles included in this meta-narrative, which were mostly published in journals with a professional health care readership, there was little semantic homogeneity about the ways in which gratitude was characterized or analyzed. Some mentioned that it was an indicator of satisfaction, others of recognition or empowerment. However, the narratives were all concerned with “care” and the role of gratitude as a qualitative factor in delivering good care.

## Discussion

### Summary of Findings

Figure 1 presents an integrated account of the reviewed literature. It maps how the six meta-narratives relate to one another, as well as to the domains of ethics, psychology, and health care research. In addition, it charts the focus and disciplinary emphasis for all included articles and demonstrates their integration into one or more meta-narratives.

It is evident that there are multiple, complex strands in the growing body of literature exploring gratitude in health care. The impact of the groundwork laid by Emmons & McCullough is considerable—33 articles in our review cited their work—but this has not led to conceptual homogeneity, and indeed, it might be unrealistic to expect this given the array of disciplines that take an interest in gratitude.

Certain themes were prominent across meta-narratives. The norm of reciprocity featured strongly in the “social capital” and “gifts” meta-narratives. In “social capital,” reciprocity was mostly appreciated as a driver of prosocial behavior, but was also criticized for locking those beholden to others’ goodwill into a cycle of perpetual, obligatory gratitude. The problems with obligatory reciprocity are also explored in the “gifts” meta-narrative where culturally accepted practices can become pernicious when they become exploitative and exacerbate health inequalities. These tensions were elaborated on in studies assigned to the “care ethic” meta-narrative, many of which explored gratitude in the context of global health and humanitarianism. The bringing together of this research illuminated a contradiction that sits unresolved in academic approaches to gratitude: the “economy” metaphors that are theory constitutive contradict the communal, moral generosity at the heart of gratitude which flinches from the obligatory reciprocity that economic metaphors demand.

Gratitude as advantageous to care givers and recipients was a theme evident in most of the articles, particularly in the “benefits,” “staff well-being,” and “quality of care indicator” meta-narratives. Some authors were forthright about how gratitude could be instrumentalized, either in eliciting prosocial behavior or in devising interventions judged likely to have beneficial psychological effects on participants. Research situated in the paradigm of positive psychology authorizes a favorable conceptualization of gratitude, but research aligned with other meta-narratives suggests that researchers should remain attuned to alternative, less affirming interpretations of situations in which gratitude is the expected response. Indeed, it may be insensitive to insist that people should find reasons to be grateful in the face of adverse life events or unsatisfactory working environments.

### Comparisons With Existing Literature

The review we have conducted complements and extends the scoping review by Aparicio et al. (2019) of gratitude between patients and their families and health professionals. Their thematic analysis of 32 publications, identified through a search using the terms “gratitude” and “health professionals,” concluded that professionals’ well-being is likely to be enhanced if they are the recipients of gratitude and called for more research. In contrast to our review, however, they do not identify any downsides to gratitude, framing it as an indicator of excellent care and a meaningful form of feedback.

The review of gratitude and health by Jans-Beken et al. (2019) focuses on experimental studies on the effects of gratitude on mental and physical health. Our findings, particularly from literature considered in the “benefits” and “staff well-being” meta-narratives, align well with their conclusion that gratitude is beneficially, although modestly, linked to social, emotional, and psychological well-being. A meta-analytic review of associations between gratitude and prosociality by Ma et al. (2017) found that gratitude plays a central role in reciprocal behaviors, which were echoed by the findings in our “social capital” meta-narrative.

### Limitations

Searching for the term “gratitude” is likely to be fallible. Lambert et al. (2009) found that a great many features are associated with gratitude, for example, appreciation, thankfulness, generosity, and graciousness. By restricting “gratitude” to titles, we effectively focused the e-search but this may be at the expense of articles which approached the topic less directly. As with most literature reviews, there is a degree of subjectivity in applying sifting criteria and other researchers might make different choices. It is possible that relevant articles are published in journals not covered by the databases we searched, and a further limitation is a publication bias for articles in English. The meta-narratives offered here did not directly “emerge” from the literature but were created through discussion among the review team. The constructions of others may differ, as might their attributions of focus and disciplinary alignment. We offer our interpretation as part of an ongoing dialogue on the relevance of social elements of communication in health care rather than a definitive account.

## Conclusion and Recommendations

The study of gratitude—its properties, implications, and effects—has been of long-standing and intense interest to a diverse range of researchers. Its general literature is vast and amorphous which can be daunting for those hoping to make a meaningful contribution to the field. This review offers a map to those hoping to find purchase in the progressive programs in which gratitude research currently finds itself. A usual recommendation for reviews of this type is to call for more systematic, evidence-based studies. However, on the basis of this review, we call for more attention to what constitutes robust “evidence”: are we content with extrapolating from responses to questionnaires that take mere seconds to complete, or should we be putting greater store in qualitative research in which responses are less constrained and more considered? Given the contested conceptual basis for gratitude, we recommend that future work should focus on understanding the way gratitude acquires meaning in real-world situations as a precursor to devising more sophisticated empirical enquiries.

The focus on health care is timely and relevant, as it becomes increasingly evident that civility in workplace culture has a definitive effect on retention, job satisfaction, and patient safety (see, for example, Armstrong, 2018; Rajamohan et al., 2019). We found relatively little attention paid to gratitude as a component of civility in care settings (addressed indirectly in Mullin, 2011 and Riskin et al., 2019), and this could usefully be explored in further research. The Covid-19 pandemic provides new opportunities for investigating gratitude. Collective expressions of appreciation for health care workers in many parts of the world have been accompanied by increasingly politicized conversations in the mainstream and social media about what constitutes meaningful gratitude. Valuable insights could be gleaned about how gratitude intersects with issues of esteem, community cohesion, and the languages of valorization that often accompany expressions of gratitude.

Sociologist Arthur Frank reminds us that the foremost task of responding to illness and disability is to increase the generosity with which we offer medical skill, and that to be generous we need to “first feel grateful” (Frank, 2004, p. 142). Given its importance to the prosocial enterprise that is health care, the challenge posed by the traits and multiple states of gratitude should encourage rather than deter the assiduous researcher. This meta-narrative review shows that research in gratitude in health care has significant potential for developing understandings of conceptual issues around the intrinsic nature of recognition and appreciation in care-giving relationships. On the evidence of this review, gratitude should be recognized as integral to the social relations that significantly influence what people think, feel, say, and do in relation to health care.

## Supplemental Material

Metanarrative_Table_Revised_Supplementary_File – Supplemental material for Gratitude in Health Care: A Meta-narrative Review Supplemental material, Metanarrative_Table_Revised_Supplementary_File for Gratitude in Health Care: A Meta-narrative Review by Giskin Day, Glenn Robert and Anne Marie Rafferty in Qualitative Health Research

Supplementary_figure_1 – Supplemental material for Gratitude in Health Care: A Meta-narrative Review Supplemental material, Supplementary_figure_1 for Gratitude in Health Care: A Meta-narrative Review by Giskin Day, Glenn Robert and Anne Marie Rafferty in Qualitative Health Research
