# Supplementary material for: Gratitude in Health Care: A Meta-narrative Review
Source: Qual Health Res. 2020 Sep 13;30(14):2303–15. doi: 10.1177/1049732320951145 (PMC7649920; doi:10.1177/1049732320951145)

Supplementary Figure 1. Overview of process for retrieval, screening and selection of articles for metanarrative review of gratitude in healthcare.

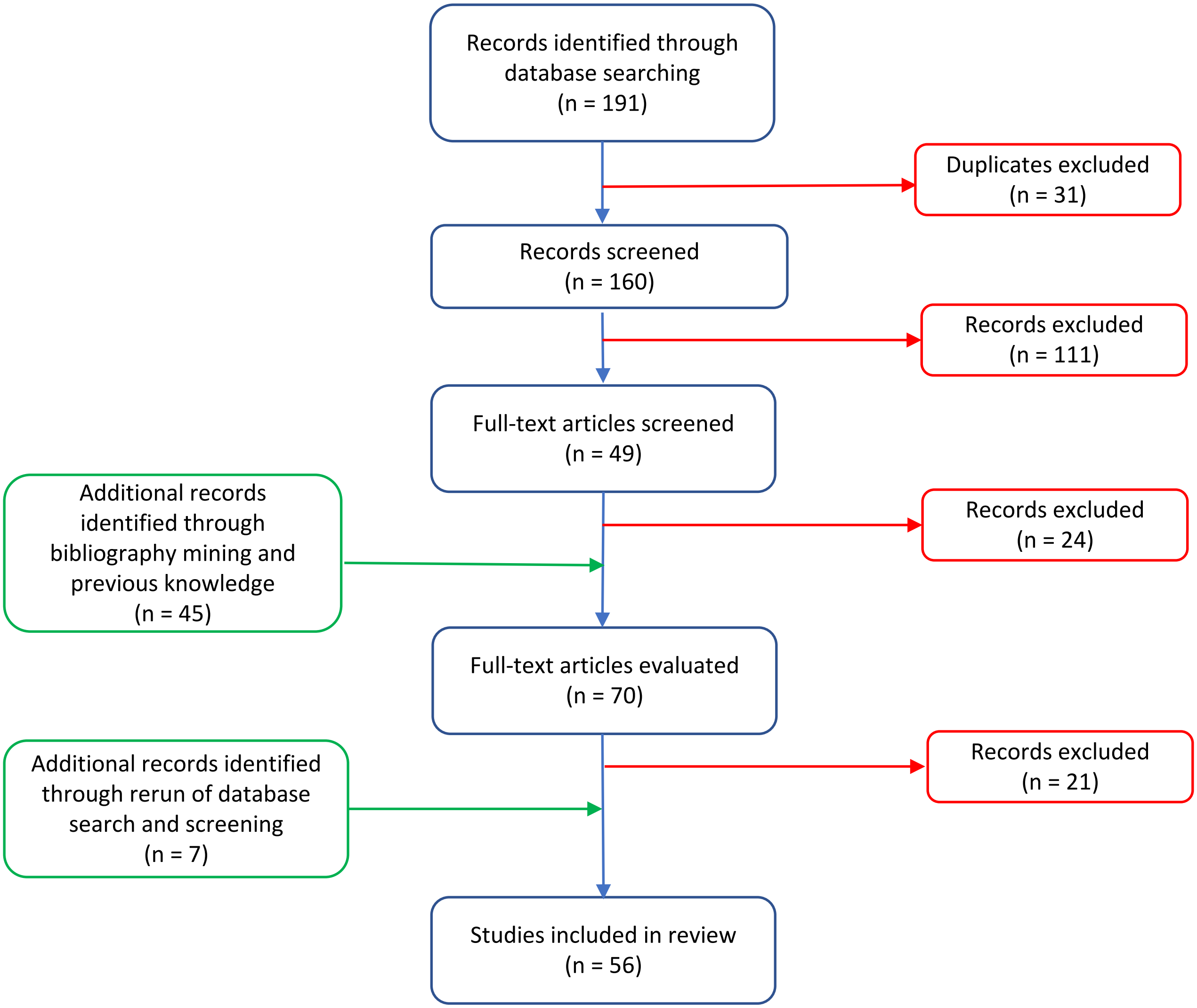

Supplement: Supplementary_figure_1 – Supplemental material for Gratitude in Health Care: A Meta-narrative Review [file Supplementary_figure_1.pdf]
